# New insights into mesenchymal stem cells in inflammatory subtypes of asthma

**DOI:** 10.3389/fimmu.2025.1649597

**Published:** 2025-08-26

**Authors:** Zheng-yan Hou, Yu-qiu Hao, Lin Zhang, Wei Li, Peng Gao

**Affiliations:** Department of Respiratory & Critical Care Medicine, Hospital 2, Jilin University, Changchun, China

**Keywords:** asthma, inflammation, mesenchymal stem cells, exosomes, immunomodulation, cell-based therapy

## Abstract

Asthma is a heterogeneous disease characterized by chronic airway inflammation, heightened reactivity, and structural remodeling. The responses of different phenotypes to traditional corticosteroid therapy vary significantly, with steroid resistance in low T-helper type 2 asthma remaining an urgent clinical challenge. In recent years, mesenchymal stem cells (MSCs) and their exosomes—mesenchymal stem cell-derived extracellular vesicles (MSC-EVs)—have emerged as promising therapeutic agents due to their potent immunomodulatory properties. In this review, we systematically explain how MSCs and MSC-EVs inhibit airway inflammation in asthma through multi-target immunoregulation, highlight their therapeutic potential in steroid-resistant asthma, and outline the challenges and optimization strategies involved in clinical translation, thereby providing a theoretical foundation for the development of novel therapies.

## 1 Introduction

Asthma is a common respiratory disorder characterized by chronic airway inflammation, heightened airway responsiveness, and structural remodeling (1–5). Its pathophysiology involves numerous inflammatory cells and mediators (6–10). Based on inflammatory cell profiles in induced sputum, asthma can be classified into four types: eosinophilic asthma, neutrophilic asthma, mixed granulocytic asthma and paucigranulocytic asthma (11). T-helper type 2 (Th2)-type asthma is characterized by activation of type 2 inflammatory pathways and is characterized by eosinophilic inflammation (12). In contrast, non-Th2 asthma lacks markers of type 2 inflammation and is characterized by neutrophilic, paucigranulocytic, or mixed inflammation (12). Corticosteroids are essential in asthma management; however, low Th2 asthma often exhibits steroid resistance and remains difficult to treat (13–17). Managing low Th2 asthma remains a significant clinical challenge in respiratory medicine. Several new biologic therapies have recently emerged. Anti-immunoglobulin E (anti-IgE) monoclonal antibodies (mAbs), such as omalizumab, reduce exacerbations by lowering free IgE levels and may delay disease progression (18). Anti-interleukin-5/interleukin-5 receptor alpha (anti-IL-5/IL-5Rα) agents, such as mepolizumab and benralizumab, effectively eliminate eosinophils, reduce acute exacerbation rates, and improve lung function (19, 20). IL-4Rα inhibitors, such as dupilumab, significantly improve mucus secretion and airway remodeling by blocking IL-4/IL-13 signaling. These are suitable for moderate to severe asthma (21). Anti-thymic stromal lymphopoietin mAbs, such as tezepelumab, inhibit upstream alarmin pathways and demonstrate efficacy across various asthma phenotypes (22). However, challenges remain in the clinical application of these drugs. Certain asthma subtypes—such as neutrophilic and mixed granulocytic asthma—respond poorly to treatment (23–25). Other concerns include disease rebound after discontinuation, complications associated with long-term use, and high treatment costs (26–29). Recent studies have found that mesenchymal stem cells (MSCs) have significant therapeutic effects in asthma, offering new avenues for treatment development.

**Figure 1:**
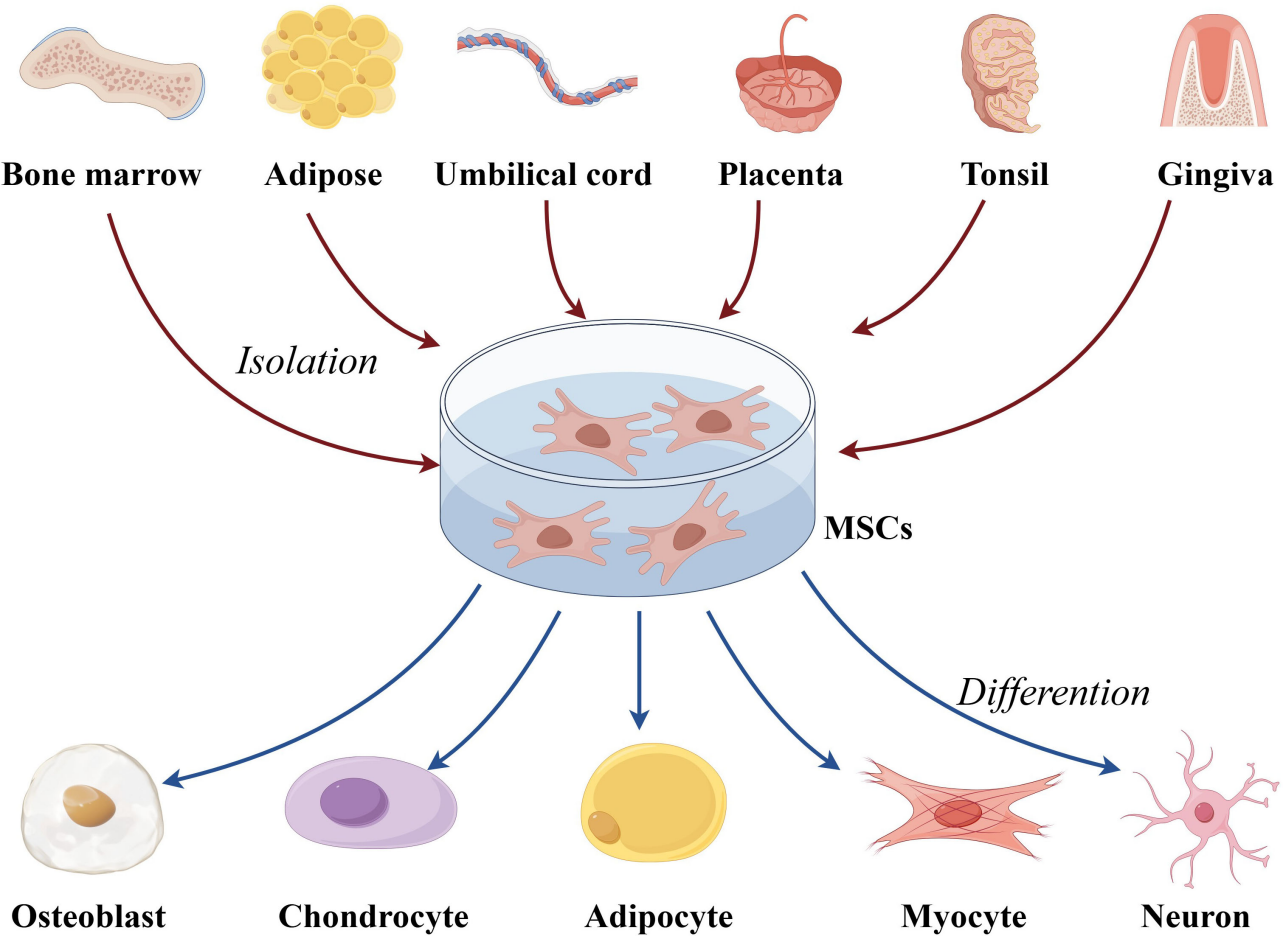
The overview of multipotentiality and multi-lineage differentiation of MSCs. MSC, mesenchymal stem cells.

MSCs are adult stem cells capable of self-renewal and multi-lineage differentiation (30–33). First identified in 1976, MSCs have since been found in nearly all human tissues, including bone marrow-derived MSCs (BM-MSCs), adipose-derived MSCs, and umbilical cord blood-derived MSCs (UC-MSCs) (34–38) (**Figure 1**). Originating from the mesoderm, MSCs exhibit a strong capacity for self-renewal and diverse differentiation potential (39–41). Extensive studies have demonstrated that MSCs possess potent immunomodulatory properties and can migrate to sites of inflammation and tumors (42). MSCs have shown remarkable therapeutic benefits for numerous diseases due to their ability to differentiate into various cell types (43–47). *In vitro*-expanded MSCs have been applied in treating a range of immune and inflammatory conditions, including autoimmune diseases and organ failure, such as Crohn’s disease of the bowel and graft-versus-host disease (38).

Recently, the potential role of MSCs in asthma has attracted increasing interest from researchers (48). However, the clinical application of MSCs faces several challenges, including the need for large cell doses, variability in cell quality, low post-transplantation survival rates, unintended effects following intravenous injection, and limited ability to cross physiological barriers such as the blood–brain barrier (49–53). Therefore, increasing attention has been directed toward extracellular vesicles (EVs) derived from MSCs and their ability to modulate normal and pathological cellular processes, including inflammation and tissue injury responses (54). MSC-derived extracellular vesicles (MSC-EVs) are lipid-based vesicles secreted by MSCs and include exosomes, microvesicles, and apoptotic bodies (55–57). Exosomes are formed through the endosomal-multivesicular body pathway, microvesicles are generated via direct budding from the plasma membrane resulting from localized membrane remodeling and shedding, while apoptotic bodies are produced through programmed cell apoptosis (58–60). Several studies have investigated the mechanisms by which MSC-EVs influence immune cell function (54, 61, 62). These vesicles carry a wide array of signaling molecules from their parent cells, such as proteins, lipids, surface receptors, enzymes, cytokines, metabolites, and nucleic acids (63, 64). By modulating innate and adaptive immune responses, MSC-EVs help regulate immunity by suppressing the activity of T cells, B cells, natural killer cells, innate lymphoid cells, macrophages, and dendritic cells (DCs), thereby influencing various asthma phenotypes (65, 66).

## 2 MSCs/EVs and asthma

MSCs and MSC-EVs exert significant effects on asthma and have emerged as promising therapeutic agents for its treatment (67–69). The therapeutic potential of MSCs in asthma largely stems from their immunomodulatory properties, which help regulate the balance between Th1 and Th2 cytokines, thereby reducing airway inflammation and remodeling (53, 70–72). Numerous studies have confirmed the immunomodulatory effects of MSCs (52, 73, 74). MSCs can reduce T cell proliferation (75, 76), inhibit the differentiation of monocytes into pro-inflammatory macrophages and DCs (77, 78), suppress the cytotoxicity and proliferation of natural killer cells (79), and limit B cell maturation and antibody production (80). These findings suggest that MSCs may influence asthma progression by modulating multiple immune cell types. MSC-EVs are increasingly recognized for their similar immunoregulatory roles and their advantages in transport and application (81). **Table 1** categorizes and summarizes asthma animal models using MSCs or MSC-EVs as a therapeutic approach.

**Table 1:** Summary of animal studies on MSC therapy for asthma.

Classification	Author	Type of cases	Asthma subtype	MSC source	Administration route	MSC dosage	Main findings	References
MSCs	Fernanda F. Cruz et al.	AHE induced- C57BL/6 mice	Mixed	BM-MSCs	IV	1×106 cells	IL-4, IL-5, IL-13,IL-6, IL-17a ↓, IFNγ ↑, lymphocytes, neutrophils, eosinophils in BALF ↓	(67)
	Geng Lin et al.	HDM induced-BALB/c mice	Mixed	HGF-DPSCs	IV	2×106 cells	IL-4, IL-5, IL-13, IgE ↓; Ckb8–1 protein expression ↓; CD4+ T cell, CCR1+ T cell proportion ↓; AHR ↓; CD4+ T cells ↓	(68)
	Fatih Firinci et al.	OVA induced-BALB/c mice	Eosinophilic	BM-MSCs	IV	1×106 cells	IgE, IL-4 ↓; goblet cells, mast cells↓; airway remodeling ↓; eosinophils in blood and BALF ↓	(69)
	Julie E Trzil et al.	Chronic allergic asthma induced cats	Eosinophilic	AD-MSCs	IV	0.36–2.5×10^7^ MSCs	LA and BWT scores ↓	(71)
	Si-Yuan Ma et al.	OVA induced-BALB/c mice	Eosinophilic	iPSC-MSCs	IV	1×10^8^ cells/kg	OVA-IgG1, OVA-IgE↓; IL-4, IL-13, IL-5 in BALF ↓; eosinophils in spleen ↓	(72)
	Guan Nan Tang et al.	OVA induced-BALB/c mice	Eosinophilic	BM-MSCs	IV	1×106 cells	IL-4, IL-5, IL-13↓; Treg cells ↑; TLR3 mRNA ↑; IFN-γ mRNA ↑; IL-12a mRNA ↑	(96)
	Ahmadi M	OVA induced-Wistar rats	Eosinophilic	BM-MSCs	IV	2×106 cells	IL-4↓, IL-13↓, IL-10↑	(97)
	Yingying Li	OVA induced-BALB/c mice	Mixed	UC-MSCs	IV	1×107 cells	IL-10 ↑, IL-17 ↓; Foxp3 ↑, RORγ ↓; Treg cells ↑, Th17 cells ↓; AHR, AI↓	(102)
	Jae Woo Shin et al.	A. alternata or HDM/DEP induced-BALB/c mice	Eosinophilic or mixed	hUC-MSCs	IV	1.0×10^5^ cells	IL-13↓; AHR↓; ILC2s↓; eosinophils in BAL↓	(106)
	Hai-Feng Ou-Yang et al.	OVA induced-C57BL/6 mice	Eosinophilic	BM-MSCs	IV	2×106 cells	IL-4, IL-5, IL-9 in BALF↓; IFN-γ in BALF ↑; Th1/Th2 ratio ↑; Total inflammatory cells, eosinophils in BALF ↓; Mast cell degranulation ↓; AHR↓	(107)
	Faouzi Braza	HDM induced-BALB/c mice	Mixed	BM-MSCs	IV	5×10^5^ cells	IL-4, IL-5, IL-13, IL-17 ↓, IL-10, IFN-γ↑; M2 macrophage polarization ↑; AHR ↓	(131)
	Abreu, S.C. et al.	OVA induced-C57BL/6 mice	Mixed	BM-MSCs	IV	1×10^5^ cells	IL-4, IL-13, TGF-β, VEGF↓; IL-10, IFN-γ↑; M2 macrophage polarization↑; eosinophils, neutrophils, BALF total cells↓; parenchymal collagen, airway remodeling↓.	(135)
	Xiaolian Song et al.	OVA induced-BALB/c mice	Eosinophilic	BM-MSCs	IV	1×106 cells	Eosinophils in BALF↓; IL-4, IL-5, IL-13↓; Alveolar macrophage M2 polarization ↑	(136)
	Qiannan Fang et al.	OVA induced-C57BL/6 mice	Mixed	GMSCs	IV	2×106 cells	CD11b+ DCs, CD11c+DCs proinflammatory↓; Th2 cell differentiation↓; Eosinophil infiltration↓; IL-5-secreting CD4+ T cells↓; HGF-dependent immunomodulation↑	(152)
	Kambiz Moghaddasi et al.	OVA induced-BALB/c mice	Eosinophilic	BM-MSCs	IV	1×106 cells	IgE, IL-4 ↓; goblet cells,mast cells↓; airway remodeling ↓; eosinophils in blood and BALF ↓	(153)
	Ligia Lins de Castro et al.	OVA induced-BALB/c mice	Eosinophilic	AD-MSCs	IV	1×106 cells	CD3+CD4+ T cells ↓; Collagen deposition ↓; TGF-β ↓; IL-5 in lung tissue↓	(155)
	Ligia L. Castro et al.	HDM induced-C57BL/6 mice	Eosinophilic	AD-MSCs	IV	1×10^5^ cells	IL-4, IL-13, TGF-β↓; CD4+ T-cells, Eosinophils↓; Collagen fiber content↓	(156)
	Nemeth K	Ragweed induced-C57BL/6 mice	Eosinophilic	BM-MSCs	IV	ns	IL-4, IL-5, IL-13 ↓; IgG1, IgE↓; eosinophil infiltration↓; mucus production↓	(158)
	Shao Lin Zeng et al.	OVA induced-BALB/c mice	Eosinophilic	BM-MSCs	IV	1×106 cells	DC maturation (CD40/CD80/CD86) ↓; IL-4, IL-5, IL-13↓; CCL17/CCL22 ↓	(176)
	Khang M. Duong	HDM induced-BALB/c mice	–	BM-MSCs	IV	1×106 cells	IL-5, IL-13↓, IL-25 ↓; Activated CD11c+ DCs, activated CD11b+ DCs ↓; AHR ↓	(178)
	J. Z. Kitoko et al.	HDM induced-C57BL/6 mice	Mixed	BM-MSCs/AD-MSCs	IT	1×10^5^ cells	IL-10 ↑; Eosinophils, B cells in BALF↓;	(186)
	Yin Yao et al.	OVA induced-mice (specific strain not specified)	Eosinophilic	iPSC-MSCs	IV	ns	IL-4, IL-5, IL-13 in BALF↓; Serum IgE ↓; IL-33 and TSLP in lungs ↓; Eosinophils, lymphocytes, neutrophils↓	(212)
	Xin-peng Han et al.	OVA induced-BALB/c mice	Eosinophilic	BM-MSCs	IV	ns	Eosinophils↓; IL-4, IL-5, IL-13, TGF-β1↓; TAK1↓; p38MAPK↓; collagen density↓; smooth muscle thickness↓	(223)
	Shu-Bin Fang et al.	OVA+LPSinduced-C57BL/7 mice	Neutrophilic	Human iPSC-MSCs	IV	1×106 cells	Neutrophils in BALF↓; Th17 cells↓; IL-17A↓; p-STAT3↓; airway inflammation score↓	(237)
	Jiling Ren	OVA induced-BALB/c mice	Eosinophilic	hUC-MSCs exosomes	IN	50 μg	IL-10↑,IL-4, IL-5, IL-6, TNF-α↓; OVA specific IgE↓; Eosinophils, Total cells in BALF↓	(66)
MSC-EVs	Liyang Dong et al.	OVA induced BALB/c mice	Mixed	hUC-MSC-derived EVs	IV	40 ug	IL-4, IL-13 in BALF↓; Collagen deposition↓; α-SMA↓; Total inflammatory cells, Eosinophils in BALF↓	(154)
	Weifeng Gu et al.	OVA induced-BALB/c mice	Eosinophilic	hUCMSC-derived migrasomes	IV	100 μg	IL-4, IL-5, IL-13 ↓; Th2 cells, eosinophils ↓;	(177)

↑or↓, significant increase or decrease; ns, not significant; AD-MSC, adipose-derived mesenchymal stem cells; AHE, aspergillus hyphal extract; AI, asthma index; α-SMA, αsmooth muscle actin; BALF, bronchoalveolar lavage fluid; BM-MSC, bone marrow-derived mesenchymal stem cells; BWT score, bronchial wall thickening score; CCR1+, C-C chemokine receptor type 1 positive; CD4+, cluster of differentiation 4 positive; Ckb8-1, chemokine beta 8-1; DCs, dendritic cells; Foxp3, forkhead box P3; EVs, extracellular vesicles; GMSCs, gingival mesenchymal stem cells; HDM, house dust mite; HGF-DPSCs, human HGF-overexpressing dental pulp stromal cells; HGF, hepatocyte growth factor; hUC-MSCs, umbilical cord-derived mesenchymal stem cells; IFN, Interferon; IgE, immunoglobulin E; ILC2s, group 2 innate lymphoid cells; IN, intranasal; IT, intratracheal; IV, intravenous; IFN-γ, interferon-gamma; IL, interleukin; IV, intravenous; iPSC-MSCs, Induced pluripotent stem cell-derived mesenchymal stem cells; LA score, lung attenuation score; p38MAPK, p38 mitogen-activated protein kinase; p-STAT3, phosphorylated signal transducer and activator of transcription 3; RORγ, RAR-related orphan receptor γ; TAK1, transforming growth factor-β-activated kinase 1; TLR3, toll-like receptor 3; mRNA, messenger RNA; TSLP, thymic stromal lymphopoietin; MSC, mesenchymal stem cells; MSC-EVs, mesenchymal stem cells-derived extracellular vesicles; TNF-α, tumor necrosis factor-alpha; Treg, regulatory T cell; UC-MSC, umbilical cord-derived mesenchymal stem cells; VEGF, vascular endothelial growth factor.

## 3 MSCs and immune and non-immune cells in asthma

**Figure 2:**
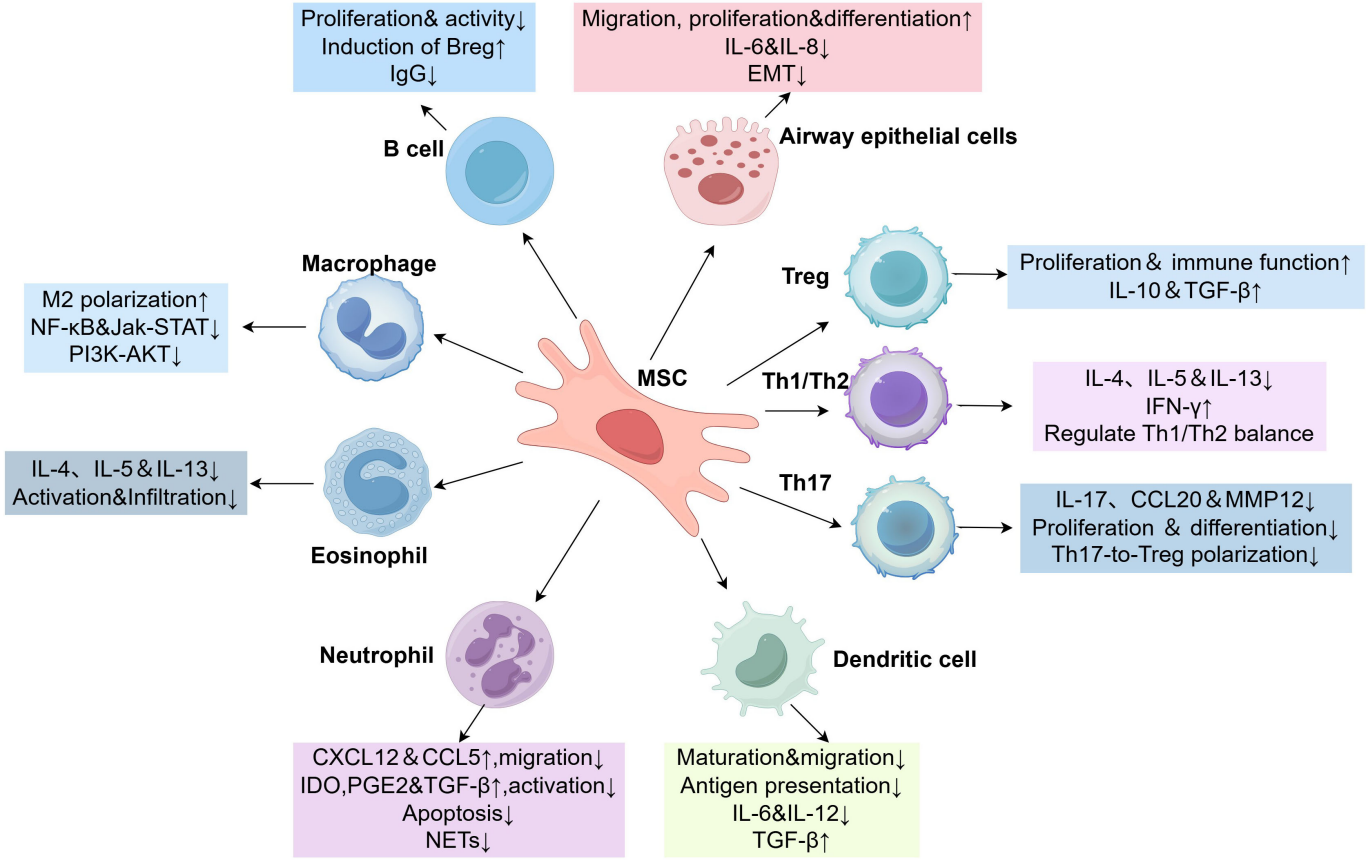
MSCs crosstalk with immune and non-immune cells. ↑or↓, significant increase or decrease; Breg, regulatory B cells; CCL, C-C motif chemokine ligand; CXCL, C-X-C motif chemokine ligand; EMT, epithelial-mesenchymal transition; IL, interleukin; Jak-STAT, janus kinase-signal transducer and activator of transcription; Treg, regulatory T cells; MSC, mesenchymal stem cells; MMP12, matrix metalloproteinase-12; NF-κB, nuclear factor kappa-B; IFN-γ, interferon-γ.

MSCs are involved in the pathogenesis of asthma through their effects on a variety of immune and non-immune cells (**Figure 2**). In this review, we summarize the molecular mechanisms of MSCs as well as the role of MSC-EVs in asthma and their therapeutic potential.

### 3.1 T cells

T lymphocyte subsets play a key role in the pathogenesis of asthma. The differentiation of various T cell subtypes and the cytokines they secrete are critical in driving different forms of asthma-related inflammation. MSC-EVs exert strong immunomodulatory effects and can regulate T cell metabolism, further influencing distinct asthma phenotypes (75, 82). Th2 cells are major drivers of eosinophilic asthma. They secrete type 2 cytokines such as IL-4, IL-5, and IL-13, which promote eosinophil proliferation and cause airway inflammation and damage (83). In contrast, Th17 cells are implicated in neutrophilic asthma. Cytokines such as IL-17, secreted by Th17 cells, strongly promote the recruitment and activation of neutrophils, resulting in increased neutrophilic infiltration in the airway, the release of inflammatory mediators and proteases, and consequent airway epithelial injury and remodeling (84, 85). MSCs can regulate various T cell states—including immature, naive, mature, effector, and memory T cells—through multiple mechanisms (86). For example, indoleamine 2, 3-dioxygenase (IDO), a rate-limiting enzyme in tryptophan catabolism induced by inflammatory cytokines, plays a role in inhibition of intracellular pathogen replication and immune regulation (87). IDO suppresses T cell proliferation and promotes regulatory T cell (Treg) differentiation by converting tryptophan to kynurenine (88). BM-MSCs have been shown to significantly upregulate IDO expression, inhibit Th17 cell differentiation, and reduce IL-17 secretion in inflammatory environments (88).

#### 3.1.1 Regulatory T cells

Tregs are a subset of T cells with significant immunosuppressive functions, characterized by the expression of forkhead box P3 (FOXP3), CD25, and CD4 (89, 90). Tregs play a critical role in asthma by suppressing excessive immune responses and reducing airway inflammation. Studies have shown that MSC-EVs can promote the differentiation of CD4+, CD25+, and FOXP3+ Tregs (91–93). This effect is mediated by specific microRNAs (miRNAs) contained in the vesicles, including miR-1470 and miR-21-5p. miR-1470 upregulates the expression of cyclin-dependent kinase inhibitor 1B (p27^Kip1^), while miR-21-5p participates in regulating Treg differentiation and function via the phosphoinositide 3-kinase (PI3K)/AKT pathway (91, 94–97). In addition, MSC-EVs can enhance the expression of miR-146a, a miRNA with significant immunomodulatory activity (98). miR-146a promotes the production of anti-inflammatory cytokines, such as IL-10 and transforming growth factor-beta (TGF-β), thereby enhancing the inhibitory function of Tregs (98). miR-126a also contributes to MSC-EV-mediated promotion of Treg differentiation. Studies have shown that MSCs can convert conventional T cells into Tregs by modulating miR-126a expression (99). miR-126a also plays a role in the stabilization of FOXP3 expression, which is a key transcription factor for Treg development and function (99). MSC-EVs can further upregulate p27^Kip1^ and down-regulate cyclin-dependent kinase 2 levels, leading to T cell cycle arrest and reduced T cell proliferation—an important immunosuppressive mechanism (100). Studies demonstrated that MSC-EVs modulate cytokine secretion in T cells. In a synovitis model, EV treatment reduced IL-17 levels and increased IL-10 and TGF-β expression (101, 102). It is important to note that MSC sources vary greatly in their ability to induce Tregs. An *in vitro* study comparing BM-MSCs and UC-MSCs found that UC-MSCs ability to induce Tregs was significantly greater than that of BM-MSCs (91). In terms of treatment, Zhang et al. reported that MSC-EVs improved disease symptoms by inhibiting T cell proliferation and enhancing Treg differentiation, leading to a decrease in proinflammatory cytokines and elevated anti-inflammatory cytokines (103).

#### 3.1.2 Th1/Th2

The imbalance between Th1 and Th2 cells is a key factor in the pathogenesis of eosinophilic asthma. By secreting TGF-β1, MSCs suppress the expression of GATA-binding protein 3 and inhibit Th2 cell proliferation (104, 105). At the same time, some studies have shown that the therapeutic effect of MSCs on severe asthma by inhibiting Th2 cells depends on the route of administration, and the dose and time need to be optimized (106, 107). Cho et al. demonstrated that MSCs reduce Th2-related cytokines, including IL-4, IL-5, and IL-13, while increasing the Th1-related cytokine interferon-gamma (IFN-γ), thereby inhibiting Th2 differentiation and restoring the Th1/Th2 balance (108, 109). Another study found that systemic injection of BM-MSCs during antigen sensitization promoted a Th1 phenotype in antigen-specific CD4 T lymphocytes, suppressing Th2-mediated allergic airway inflammation through an IFN-γ-dependent mechanism (110). Interestingly, Chen et al. reported that MSC-EVs may promote the transformation of Th1 to Th2 cells, contrary to the findings above. However, that study primarily noted that the proportion of Th1 cells was significantly decreased and that of Th2 cells was significantly increased following MSC-EV treatment, without investigating the underlying molecular mechanisms in detail (111). In addition, studies have shown that MSCs can inhibit the phosphorylation of STAT3 by upregulating the expression of protein tyrosine phosphatase non-receptor type 2, thereby reducing the production of IFN-γ and IL-4 by Th1 and Th2 cells, and suppressing their differentiation and proliferation (112, 113). Combined with the findings of Cho et al., the effect of MSCs on IFN-γ appears to be bidirectional—inhibiting its production and signaling and promoting its expression and functional enhancement. The exact mechanism remains unclear and may depend on the specific biological environment and regulatory pathways involved.

#### 3.1.3 Th17

Th17 cells secrete cytokines such as IL-17 and IL-12, promoting inflammatory responses and airway remodeling, and are thought to contribute to glucocorticoid-insensitive asthma subtypes, such as neutrophilic asthma (84, 114, 115). It has been demonstrated that treating rats with experimental autoimmune uveitis using MSCs significantly reduced the production of Th17-related cytokines, such as IL-17, lowered the proportion of Th17 cells among lymphocytes, and inhibited interleukin differentiation and activity (116). Guan et al. suggested that adipose-derived stem cells (ADSCs) directly inhibit Th17 cell proliferation through contact mediated by programmed death-ligand 1 (PD-L1) on their cell surface, and also secrete TGF-β and prostaglandin E2 (PGE2) to suppress Th17 cells (117). RNA sequencing analysis revealed that ADSCs significantly downregulated Th17-associated genes, including *IL17A*, *CCL20*, and *MMP12* (117). Pathway analysis revealed that ADSC treatment significantly inhibited the activation of IL-17 signaling pathway (117). Other studies reported that gingiva-derived MSC-EVs reduced NF-κB phosphorylation by suppressing IKKB expression via miR-148a-3p, thereby inhibiting the IKKB/NF-κB signaling pathway, reducing IL-17A levels, increasing IL-10 production, and ultimately suppressing Th17 cell proliferation and differentiation while alleviating inflammation (118, 119). MSCs also regulate Th17 cell metabolism through mitochondrial transfer. Studies have shown that tunneling nanotubes and EVs can transfer mitochondria to Th17 cells, leading to metabolic reprogramming. After MSC reprogramming, Th17 cells exhibited increased oxidative phosphorylation and reduced glycolysis through the mTORC1 pathway, which inhibited the proliferation and function of Th17 cells. Moreover, the expression of *FOXP3* in Th17 cells was significantly increased following mitochondrial transfer, suggesting a transition toward Treg cell phenotype (120, 121).

### 3.2 Monocytes and macrophages

In asthma, macrophages are key immune cells involved in phagocytosis, the release of inflammatory mediators, antigen presentation, and the regulation of immune responses (122, 123). Macrophages can be classified into two main phenotypes: classically activated (M1) and alternatively activated (M2) macrophages (124). The balance between M1 and M2 macrophages is particularly important in asthma pathogenesis. M2 macrophages are often associated with the promotion of airway inflammation and tissue remodeling (125). M1 macrophages are activated by Th1 cytokines, such as IFN-γ, and by toll-like receptor (TLR) signaling. Upon activation, M1 macrophages produce proinflammatory cytokines including IL-6 and IL-1β, which promote neutrophilic inflammation (126). In contrast, M2 macrophages are induced by Th2-derived cytokines, particularly IL-4 and IL-13, to produce eosinophil-promoting cytokines such as IL-10 and TGF-β (127). Paucigranulocytic asthma, a milder subtype of asthma, is characterized by a predominance of macrophages; however, its molecular mechanisms remain poorly understood (128, 129). Macrophage polarization induces Th1 and Th2 cytokine production, thereby contributing to the pathogenesis of eosinophilic asthma and neutrophilic asthma (130).

Several studies have shown that BM-MSCs can influence macrophages through different mechanisms, potentially affecting different asthma phenotypes (131). In the pathogenesis of asthma, macrophage polarization plays an important role in modulating the inflammatory response. Numerous studies have demonstrated that MSCs can promote M2 polarization (132–136). MSCs and MSC-EVs can induce the shift from pro-inflammatory M1 macrophages to anti-inflammatory M2 macrophages by secreting anti-inflammatory factors such as tumor necrosis factor-stimulated gene-6 (TSG-6) and IL-10, thereby reducing the inflammatory response (137–139). Furthermore, several studies have demonstrated that miRNAs and proteins carried by MSC-EVs—including miR-21-5p, miR-146a-5p, miR-451, miR-16, and galectin-1—can promote M2 polarization by inhibiting inflammatory pathways involving NF-κB, Jak-STAT, and PI3K-AKT, thus reducing inflammation (138, 140–142). In addition, MSC-derived mitochondria have been found to transfer to macrophages and promote M2 polarization by improving mitochondrial function (143). Li et al. demonstrated that MSCs can inhibit CD4+ T cell proliferation and promote M2 macrophage polarization in a TSG-6-dependent manner, thereby reducing inflammation (144). Through miRNA sequencing and proteomic analysis of mesenchymal stem cell-derived exosomes (MSC-Exos) and MRC-5-derived exosomes, Liu et al. proposed that MSC-Exos may inhibit pyroptosis via miRNA-mediated regulation of the caspase-1 pathway or through proteins with immunomodulatory functions. However, the specific miRNAs and proteins responsible remain unidentified (145).

When M1-type macrophages were co-cultured with MSCs, CD54-specific upregulation and enrichment were detected at the contact interface, and this specific interaction induced calcium signaling and enhanced the CD54-mediated immunosuppressive capacity of MSCs, which may represent an additional mechanism by which MSCs inhibit the inflammatory response (146).

Regarding monocytes, Du Rocher et al. demonstrated that MSCs induced phenotypic changes in monocytes, including downregulation of major histocompatibility complex class I and II, CD11c, and CCR5, and upregulation of CD14 and CD64 expression (147). These changes were accompanied by a reduction in IL-1β and IL-6 production by the monocytes. Overall, MSCs reduce the number of monocyte-derived macrophages and promote the polarization of macrophages from the M1 to the M2 phenotype, thereby alleviating airway inflammation.

### 3.3 Eosinophils

Eosinophilic asthma is a subtype of asthma characterized by elevated levels of eosinophils in the airway. It is associated with several cytokines, such as IL-4, IL-5, and IgE, and several novel immune agents have also been proposed for this asthma subtype (148–151). In recent years, numerous studies have confirmed that MSCs have significant effects on eosinophils and related cytokines (152, 153). Experiments have shown that human UCMSC-EVs treated with hypoxia can reduce eosinophilic infiltration in allergic airway inflammation and decrease levels of Th2 cytokines such as IL-4, IL-5, and IL-13 (154, 155). Ligia et al. also demonstrated that multiple infusions of fat-derived MSCs can reduce eosinophil-mediated inflammation in asthmatic models through immunosuppression (156). Interestingly, a case report suggested that intravenous MSC infusion significantly improved eosinophilic infiltrating gangrene of the lower extremity in patients with eosinophilic granulomatosis with polyangiitis, inhibiting local eosinophilic degranulation (157). Overall, MSCs can inhibit eosinophilic inflammation, and the specific mechanisms involved have been explored in recent years. For example, gingiva-derived MSCs inhibit eosinophil activation by secreting hepatocyte growth factor (152). BM-MSCs can inhibit eosinophil-related inflammation through TGF-β signaling pathway (158). In addition, cord blood MSCs have been shown to alleviate eosinophilic inflammation in atopic dermatitis and asthma by downregulating IgE and eosinophilic cationic protein levels (159). A study demonstrated that tonsil-derived MSCs may reduce allergic symptoms and eosinophilic infiltration by inhibiting the induction of cytokines such as IL-25 and IL-33, as well as chemokines such as CCL11 and CCL24 (160). Another study confirmed that MSCs can reduce total IgE, IL-5, and eosinophilic inflammatory cells through the expression and transduction of the *IL10* gene (161). Notably, MSCs can also indirectly affect eosinophils by influencing other cells. Placenta-derived MSCs can reduce eosinophil-mediated airway hyperreactivity and inflammation in asthmatic models by inhibiting Th17 cell differentiation and promoting Treg cell expansion (102). Although MSCs inhibit eosinophilic inflammation, their specific roles and mechanisms in eosinophilic asthma require further investigation.

### 3.4 Neutrophils

Neutrophilic asthma is a subtype of asthma characterized by neutrophil-dominant airway inflammation and is closely associated with refractory, severe, and fatal asthma (162). It is often difficult to treat because of resistance to glucocorticoid therapy and the lack of other effective treatments (130). The pathogenesis of neutrophilic asthma involves the recruitment and activation of neutrophils in the airway (163). Neutrophils release a variety of pro-inflammatory mediators, including cytokines, chemokines, and neutrophil extracellular traps (NETs). These mediators contribute to airway inflammation, hyperresponsiveness, and remodeling, which underlie the clinical symptoms of asthma (164–166). By secreting chemokines such as CXCL12 and CCL5, MSCs inhibit the over-recruitment of neutrophils to inflammatory sites, thereby reducing tissue damage (167). Another study demonstrated that intratracheal administration of MSCs reduced CXCL1 expression via secretion of TSG-6, which inhibited neutrophil recruitment (168). Furthermore, MSCs secrete molecules such as IDO, PGE2, and TGF-β, which directly inhibit excessive neutrophil activation, reduce pro-inflammatory cytokine release, and alleviate inflammation (169, 170). BM-MSCs have been shown to inhibit neutrophil apoptosis and reduce reactive oxygen species (ROS) production (171). Moreover, MSC treatment can reduce chemotaxis, ROS production, and NADPH oxidase activity by upregulating CD24 expression, shifting activated neutrophils toward a senescent neutrophil phenotype and thereby suppressing inflammation (172). Another possible mechanism involves NETs. NETs are web-like structures composed of decondensed chromatin and antimicrobial proteins released by neutrophils to trap and kill pathogens (173). MSCs can regulate many neutrophil functions, including migration, ROS production, and NET formation. The direction of these effects depends closely on the specific environment and the source of MSCs (174). Under non-pathological conditions, MSCs enhance neutrophil migration and ROS production, whereas in inflammatory states, MSCs suppress these functions as well as NETosis (174). EVs secreted by MSCs carry miRNAs such as miR-199, which inhibit NET formation and reduce the release of pro-inflammatory mediators, thereby attenuating inflammation (167). Wang et al. demonstrated that MSCs can reduce NET formation by inhibiting the MEK/ERK signaling pathway (175). In addition, the source of MSCs influences their effect on neutrophils: MSCs derived from healthy donors block neutrophil infiltration and NETosis, whereas MSCs derived from patients with cancer enhance these functions (174). Hypoxia-challenged MSC-derived EVs have been shown to reduce excessive NET formation, promoting diabetic wound healing (175). This effect is mediated by the transfer of miR-17-5p, which targets the TLR4/ROS/MAPK pathway (175). Targeting NETs with MSCs may represent a novel therapeutic strategy for asthma.

### 3.5 Dendritic cells

DCs play an important role in various types of asthma by regulating immune cell activation and the inflammatory response. MSCs can inhibit the maturation, migration, and antigen-presenting functions of DCs through cytokine secretion, EVs, and direct cell contact, thereby exerting therapeutic effects in asthma (176–178). A study by Saeidi et al. suggested that UC-MSCs and BM-MSCs inhibit the differentiation, maturation, and endocytosis of monocyte-derived DCs under *in vitro* conditions via secreted factors (78). Peng et al. found that MSC-EVs can inhibit the differentiation of human monocytes into DCs and downregulate the expression of CD40, CD80, CD86, and HLA-DR, although they do not affect mature DCs (mDCs) (179). Reis et al. also demonstrated that MSC-EV treatment impaired antigen uptake by immature DCs and prevented their maturation, resulting in decreased expression of CD83, CD38, and CD80, reduced secretion of IL-6 and IL-12p70 and increased TGF-β production. The study also proposed a novel mechanism whereby MSC-EVs regulate DC function by targeting the *CCR7* gene for degradation via miRNAs such as miR-21-5p, thereby significantly reducing their migration toward CCL21 (94). Another study showed that MSCs suppressed DC function by inhibiting IL-10/STAT3-mediated *CST7* gene transcription (180). Another study suggested that MSCs may regulate the MAPK signaling pathway in DCs, secrete galectin-1 (Gal-1) to upregulate Gal-1 expression in DCs, and induce the development of tolerogenic DC immunophenotypes, thereby inhibiting DC function (181). Furthermore, Xiao-Qing et al. demonstrated that small MSC-EVs inhibited the activation of mDCs on type 2 innate lymphoid cells (ILC2s) in patients with allergic rhinitis (182). Mechanistically, the PGE2-EP2/4 axis plays a key role in the immunomodulatory effects of EV-modified mDCs on ILC2s. The study also confirmed the ability of MSC-EVs to modulate DC function and inhibit the Th2 immune response in allergic conditions (182). By increasing the production of IL-10, EV-treated DCs can suppress the production of Th2 cytokines such as IL-4, IL-9, and IL-13, thereby alleviating allergic reactions (182). These findings suggest that MSC-EVs may have therapeutic potential for treating allergic airway inflammation by targeting DCs.

### 3.6 B cells

B cells play pro-inflammatory and anti-inflammatory roles in asthma by producing IgE antibodies, regulating immune responses, and participating in airway inflammation. They are key players in the immunopathogenesis of asthma. Studies by Gauvreau et al. have found elevated levels of IgE^+^ B cells in the airways of patients with asthma, suggesting their involvement in allergic inflammation (183). Beyond antibody production, B cells also act as antigen-presenting cells that regulate T cell responses. In asthma, B cells can influence the differentiation of Th2 cells, thereby contributing to allergic inflammatory processes (184). Studies have shown that MSCs can regulate B cell proliferation and differentiation while also inhibiting B cell apoptosis (80, 185, 186). Increased PD-L1 in lipopolysaccharide-treated human adipose-derived MSCs was found to inhibit B cell proliferation and IgG secretion via the NF-κB pathway (187). Guo et al. reported that MSC treatment downregulated Th17 cells and upregulated CD1d^high^CD5^+^ regulatory B (Breg) cell activity, thereby reducing the severity of experimental autoimmune encephalomyelitis (188). Epstein-Barr virus-induced gene 3 (EBI3), a β-subunit of the IL-12 cytokine family, is induced by Epstein-Barr virus infection and forms various immune-regulating cytokines by pairing with different α-subunits and play an important role in immune regulation (189, 190). Tonsil-derived MSCs have been shown to alleviate B cell-mediated immune responses and increase the population of IL-10-producing Breg cells by upregulating EBI3 expression (190). Similar to T cells, miRNAs contained in MSC-EVs also exert regulatory effects on B cells. miR-125b, derived from labial gland MSC exosomes, have been shown to influence primary Sjögren’s syndrome by directly targeting *PRDM1*. As a result, the proportion of CD19+CD20-CD27+CD38+ plasma cells decreased significantly (191). EGR2 plays a key role in B cell development, particularly during their survival and differentiation stages (192, 193). A study on neonatal hypoxia-ischemia brain injury suggested that MSC-EV-derived miR-410 may upregulate *EGR2* and *BCL2* expression by downregulating *HDAC1*. These findings suggest that miR-410 may have a regulatory role in B cell function, although further studies are needed (194). In addition, MSCs may influence asthma by modulating Bregs, a heterogeneous group of B cell subsets with immunosuppressive properties similar to those of Tregs (195). Chao et al. demonstrated that human UC-MSCs protected against experimental colitis by increasing the number of CD5^+^ Bregs and correcting imbalances between Treg/Th17/Th1 cell subsets (196). Another study also confirmed that MSCs promote the survival and proliferation of CD5^+^ Bregs (197). Multiple studies have shown that MSCs can enhance Breg production. Given the pivotal role of B cells in adaptive immune responses, further research is warranted to elucidate how MSCs modulate asthma pathogenesis through B cell-mediated mechanisms.

### 3.7 Mast cells

Mast cells (MCs) have long been recognized as key effector cells in asthma because of their increased abundance in the airways of patients with asthma compared with healthy individuals (197–199). MC activation influences the phenotype of asthma. A study by Tiotiu et al. demonstrated that IL-33-stimulated MC signatures were associated with severe neutrophilic asthma, while IgE-activated MCs were associated with eosinophilic phenotypes (200). Regarding corticosteroid responsiveness, a study by Alzahrani et al. suggested that MC-derived mediators may contribute to glucocorticoid insensitivity in severe asthma (201). MSC-derived exosomes have been shown to inhibit TLR7-mediated MC activation and reduce pro-inflammatory cytokine release (202). In addition, another study demonstrated that MSC-derived microvesicles suppressed MC activation by upregulating PGE2 production and E-prostanoid 4 (EP4) receptor expression (203). Brown et al. reported that co-culturing MSCs with MCs resulted in reduced MC degranulation, pro-inflammatory cytokine production, and chemotaxis. These effects were mediated via upregulation of COX-2 and enhanced by the activation of EP4 receptors on MCs (204). Moreover, MSCs have been shown to reduce levels of IL-5 in patients with asthma, a key cytokine for MC activation (205). Reduced IL-5 levels can lead to decreased MC activity, thereby reducing asthma symptoms (206, 207). Overall, MSCs can suppress MC function and attenuate inflammatory responses through various pathways, offering potential benefit across asthma phenotypes. Nevertheless, further studies are needed to confirm these effects.

### 3.8 Epithelial cells

Airway epithelial cells contribute to the formation and development of airway inflammation and hyperresponsiveness in asthma through their barrier function and the release of various inflammatory mediators (208, 209). MSCs can modulate epithelial cell function via the secretion of paracrine factors that promote repair by enhancing migration, proliferation, and differentiation (210). Placenta-derived MSCs have been shown to reduce the expression of inflammatory cytokines such as IL-6 and IL-8 in epithelial cells exposed to particulate matter, exerting anti-inflammatory effects that may aid asthma control (211). Another study demonstrated that MSCs reduce inflammation by forming tunneling nanotubes to transfer mitochondria into airway epithelial cells with mitochondrial dysfunction (212). Connexin 43 (CX43) is a key molecule regulating mitochondrial transfer, and inhibiting CX43 weakens the therapeutic effect (212). The epithelial-mesenchymal transition (EMT) plays an important role in airway remodeling in asthma. During EMT, epithelial cells lose their polarity and adhesion properties, acquire mesenchymal characteristics, and exhibit increased migratory and invasive capabilities (213). In patients with asthma, exposure to allergens, contaminants, and infections activates the TGF-β1/SMAD signaling pathway, inducing EMT. This results in increased TGF-β1 expression, reduced levels of epithelial markers such as E-cadherin, and upregulation of mesenchymal markers such as vimentin and alpha-smooth muscle actin (214, 215). Song et al. demonstrated that MSCs and their exosomes significantly inhibit airway remodeling and the EMT by suppressing the Wnt/β-catenin signaling pathway (216). Additional studies have provided direct or indirect evidence supporting the ability of MSCs to inhibit the EMT in bronchial epithelial cells, suggesting promising therapeutic potential for MSCs in treating airway diseases characterized by excessive EMT and fibrosis (217–219). Further research is needed to explore the role of MSCs in modulating epithelial cell responses in asthma.

### 3.9 Airway smooth muscle cells

A hallmark of asthma is airway remodeling, which is closely associated with airway smooth muscle cell (ASMC) proliferation and migration (220). Experimental studies have demonstrated that extracellular miR-301a-3p from ADSCs can significantly reduce remodeling and the inflammatory response in PDGF-BB-stimulated ASMCs by targeting the STAT3 pathway (221). Several studies have shown that MSCs or their EVs can reduce airway smooth muscle thickness and improve airway remodeling in asthmatic mouse models (222–224). However, the precise signaling pathways remain unclear to be fully elucidated. Findings from Lin et al. suggest that the PI3K/AKT signaling pathway may play a role (225). Further investigation is needed to confirm this mechanism.

## 4 Effect of MSCs/MSC-EVs on asthma subtypes

As mentioned above, asthma can be divided into four inflammatory subtypes based on induced sputum examination. The proportion of eosinophils in sputum is significantly increased in patients with eosinophilic asthma, which is closely related to Th2 immune response and responds well to glucocorticoid treatment (12, 226). The percentage of neutrophils in sputum of patients with neutrophilic asthma is significantly increased, which is related to Th1 and Th17 inflammatory pathways. Neutrophilic asthma is often seen in patients with severe asthma or patients who smoke, and shows poor response to glucocorticoids, and is often accompanied by airway remodeling (227, 228). Patients with mixed granulocytic asthma have both increased eosinophils and neutrophils in sputum, and their clinical manifestations and pathological features are more complex and their symptoms are more severe (229). The proportion of eosinophils and neutrophils in sputum of patients with paucigranulocytic asthma is not significantly increased, and the related mechanism is not clear, but may relate to airway mucus hypersecretion or epithelial barrier dysfunction; the symptoms are persistent but there is a lack of typical inflammatory markers (230–232).

MSCs and MSC-EVs influence various asthma subtypes by impacting both immune and non-immune cells. In eosinophilic asthma, MSCs and MSC-EVs can inhibit Th2 cells differentiation by blocking GATA3 expression through TGF-β1 (104, 105), and activate PI3K/AKT pathway through miR-1470 and miR-21-5p to promote Treg expansion, reducing airway inflammation (91, 97). At the same time, they also regulate macrophages, induce their polarization to the M2 phenotype by TSG-6 and IL-10 (137–139), and inhibit the NF-κB inflammatory pathway through miR-21-5p and miR-146a-5p (140–142), exerting anti-inflammatory effects by regulating macrophages. In addition, they can directly inhibit eosinophil activation through hepatocyte growth factor secretion and TGF-β signaling (152, 158). They can also block mast cell degranulation by COX-2 up-regulation and EP4 receptor activation (204), repair barrier function by CX43-mediated mitochondrial transfer (212). However, it should be noted that the curative effects of MSCs from different sources are significantly different. For example, UC-MSCs have a significantly stronger ability to induce Treg than BM-MSCs (91), and MSC-EVs may promote Th2 transformation through unknown mechanisms (111).

For neutrophilic asthma, MSCs and MSC-EVs inhibit Th17 cells differentiation through IDO-mediated tryptophan metabolism (88), block Th17 cells proliferation by PD-L1 contact (117), and promote their transformation into Treg through mitochondrial reprogramming (120, 121). They can also block neutrophil recruitment by down-regulating CXCL1 by TSG-6 (168), inhibit NET formation by miR-199 (167), and inhibit NETosis by targeting TLR4/ROS/MAPK pathway by miR-17-5p (175). This reduces neutrophilic inflammation. Activated by TLR4, M1 macrophages can highly express CD86 and other markers, secrete IL-6, IL-1β and TNF-α, and directly recruit neutrophils to infiltrate the airway (227). MSCs can regulate macrophages, inhibit M1 polarization by activating calcium signal through CD54 interaction (146), and block cell pyroptosis by caspase-1 pathway inhibition (145). Activated MCs can directly recruit neutrophils to the airway by releasing chemokines such as LTB4, or even capture and internalize neutrophils by forming an intracellular mast cell trap, using its undigested substances to enhance its pro-inflammatory function and further recruit more neutrophils (233). MSCs and MSC-EVs can block IL-33-activated MCs and attenuate the activation of MCs by inhibiting TLR7 signaling pathway (202). It is worth noting that MSCs in the inflammatory state significantly inhibit the function of neutrophils, but different sources of MSCs have opposite effects; healthy donor MSCs inhibit NETosis, while MSCs derived from patients with cancer enhance this effect (174).

In patients with mixed granulocyte asthma, not only the percentage of neutrophils in induced sputum, but also the level of exhaled nitric oxide, was significantly increased, suggesting the coexistence of type 2 and non-type 2 inflammation (229). According to the above, MSCs and MSC-EVs can inhibit mixed granulocytic asthma by inhibiting eosinophilic inflammation and neutrophilic inflammation simultaneously by different immune cells. In paucigranulocytic asthma, MSCs and MSC-EVs mainly play a role by regulating epithelial barrier function and mucus secretion. Studies have shown that the characteristic mucus hypersecretion and epithelial barrier damage of this subtype of asthma are related to the abnormal release of epithelial-derived alarmins (230–232). MiR-146a and other molecules carried by MSC-EVs can inhibit the NF-κB pathway in airway epithelial cells and reduce the expression of MUC5AC mucin (210, 216). At the same time, MSCs repair epithelial tight junction proteins and restore barrier integrity through CX43-mediated mitochondrial transfer (212). In addition, HGF secreted by MSCs can down-regulate IL-25 and IL-33 expression (152), indirectly inhibit ILC2 activation, and block the vicious cycle of non-Th2 type inflammation, thereby improving airway hypersecretion and persistent symptoms of paucigranulocytic asthma (160, 230).

## 5 MSCs and asthma treatment

Corticosteroids play a central role in asthma treatment, with inhaled corticosteroids being essential for maintenance therapy in some patients (234). However, some patients develop resistance, and those with low Th2 asthma are not sensitive to steroids (235). In recent years, studies have suggested that MSCs can be used to treat asthma by controlling airway inflammation (205, 236, 237). Numerous animal studies have shown that MSCs can regulate Th1/Th2 balance, inhibit the maturation and function of DCs, suppress B cell maturation and antibody production, reduce the infiltration and activation of eosinophils and neutrophils, and inhibit M2 macrophage polarization. These mechanisms contribute to reduced airway inflammation and hyperresponsiveness, thereby alleviating asthma symptoms. In clinical research, several MSC-based therapies have been or are currently being evaluated. However, no MSC-related asthma treatments have passed phase 3 clinical trials. One discontinued phase I clinical trial (NCT03137199) evaluated the efficacy of intravenously administered BM-MSCs in two groups of patients with asthma, assessing lung volume and function, peripheral eosinophil levels, dyspnea, and quality of life every 4 weeks. Unfortunately, this trial was terminated in 2020. Notably, in a separate phase I clinical trial, a 68-year-old male patient with asthma received an intravenous infusion of human UC-MSCs cultured *in vitro*. Follow-up at 2 and 6 months post-treatment revealed a marked reduction in asthma attack frequency and a decreased dependence on inhalers and supplemental oxygen. No treatment-related adverse events were reported, indicating that MSC therapy significantly improved the patient’s quality of life (238). Regarding the safety of MSC therapy, a phase 1 clinical study is currently being conducted to evaluate the safety, toxicity, and potential mechanisms of interferon-γ-induced mesenchymal stromal cells (γMSCs) in the treatment of moderate-to-severe persistent asthma. Participants are divided into low- and high-dose groups, and the primary outcome measures include the number of adverse events and severe adverse events post-interventions, as well as the incidence of grade ≥3 adverse reactions attributed to the γMSC product. This trial is ongoing (NCT05035862). In another ongoing clinical experiment, airway epithelial cells were obtained from participants via bronchoscopy to evaluate the effects of adult stem cell products on asthmatic airway epithelium *in vitro*. The study aims to explore the utility of MSC secretome in eliminating IgE- and type-2 cytokine-driven immune response and reversing airway remodeling (NCT04883320). MSCs show therapeutic promise for asthma; however, challenges remain. These include difficulties in storage and transportation, the risk of incomplete differentiation, and limited lifespan (239–241). Therefore, MSC-EVs may represent a more practical alternative. In a currently ongoing phase II clinical trial (NCT04602104), patients are assigned to low-, medium-, and high-dose groups to receive either aerosolized human MSC exosomes or saline for seven consecutive days. Other trials are evaluating the safety of exosomes derived from healthy volunteers (NCT04313647). To date, however, no clinical trials evaluating MSC-EV therapy for asthma have been completed, and further research is needed.

## 6 Prospects and conclusion

Overall, current evidence suggests that MSCs exert diverse and beneficial effects in asthma. MSCs can inhibit airway inflammation and remodeling—two hallmark features of asthma—indicating their promise as a potential therapeutic approach. Given the limitations of existing therapies and the urgent need for development of novel treatments, MSC-based approaches represent an area of growing interest. MSCs appear to regulate immune responses across various inflammatory subtypes of asthma by modulating the proliferation, differentiation, and cytokine secretion of several key cell types involved in the pathogenesis of asthma, such as B cells, T cells, macrophages, MCs, neutrophils, and eosinophils. However, the specific mechanisms and signaling pathways of MSCs regulation in asthma remain unknown, and warrant further investigations.

The available evidence suggests that MSCs and their derived EVs have demonstrated significant therapeutic potential in asthma by modulating immune and non-immune cell functions. They inhibit the proliferation and activation of T cells, B cells, eosinophils, neutrophils, and DCs; promote Tregs and Bregs; and induce the polarization of macrophages from pro-inflammatory M1 to anti-inflammatory M2 phenotypes. Collectively, these actions contribute to the reduction of airway inflammation and hyperresponsiveness. Although the therapeutic potential of MSCs has shown positive results in different studies as well as in clinical trials, the specific mechanisms and signaling pathways of MSCs in patients with different phenotypes of asthma are still under investigation.

MSC therapy has the potential to transform asthma treatment, particularly for patients with steroid-resistant subtypes such as neutrophilic asthma. However, one of the key limitations of MSC therapy is the relatively low survival rate of the cells and high cost because of variability in cell quality when compared with existing biologic agents and corticosteroid-based therapies. Future development of MSC-based therapies should focus on improving cell quality and survival, enhancing immunomodulatory capacity, and minimizing production costs. Pretreatment strategies—such as hypoxia or cytokine priming—may enhance the therapeutic efficacy of MSCs while simultaneously reducing the associated costs. Compared to MSCs, MSC-EVs offer greater therapeutic promise due to their enhanced safety, improved stability, and more flexible delivery options.

Research into the role of MSC-EVs in regulating immune and structural cells in asthma is still in its early stages, and more clinical trials and studies on the specific mechanism of action need to be carried out. Further investigations are needed to establish the safety, efficacy, and mechanism of action of MSCs and MSC-EVs in asthma therapy. These efforts will be critical in paving the way for novel therapeutic strategies for this chronic inflammatory disease.
